# Women, Partners, and Mothers–Migratory Tendencies of Psychiatric Trainees Across Europe

**DOI:** 10.3389/fpubh.2019.00143

**Published:** 2019-06-14

**Authors:** Mariana Pinto da Costa, Ana Giurgiuca, Eirini Andreou, Franziska Baessler, Visnja Banjac, Ewelina Biskup, Jozef Dragasek, Emam El-Higaya, Kfir Feffer, Dorota Frydecka, Juhana Kaaja, Athanasios Kanellopoulos, Ozge Kilic, Petra Marinova, Marija Mitkovic-Voncina, Rosa Molina-Ruiz, Claudia Palumbo, Maja Pantovic-Stefanovic, Iva Rakos, Maria Stoyanova, Sonila Tomori, Livia De Picker

**Affiliations:** ^1^Institute of Biomedical Sciences Abel Salazar, University of Porto, Porto, Portugal; ^2^Unit for Social and Community Psychiatry (WHO Collaborating Centre for Mental Health Services Development), Queen Mary University of London, London, United Kingdom; ^3^Hospital de Magalhães Lemos, Porto, Portugal; ^4^The “Carol Davila” University of Medicine and Pharmacy, Bucharest, Romania; ^5^Cyprus Mental Health Services, Nicosia, Cyprus; ^6^Department of General Internal and Psychosomatic Medicine, Heidelberg University Hospital, Heidelberg, Germany; ^7^Clinic of Psychiatry, University Clinical Center of the Republic of Srpska, Banjaluka, Bosnia and Herzegovina; ^8^Shanghai University of Medicine and Health Sciences Basic Medical College, Shanghai, China; ^9^Division of Internal Medicine, University of Basel, Basel, Switzerland; ^10^1st Department of Psychiatry, Faculty of Medicine, Pavol Jozef Safarik University, Kosice, Slovakia; ^11^Galway Acute Child and Adolescent Psychiatry Inpatient Unit, Merlin Park University Hospital, Galway, Ireland; ^12^Lev-Hasharon Mental Health Center Tzur-Moshe, Israel and Tel-Aviv University, Tel-Aviv, Israel; ^13^Department of Psychiatry, Wroclaw Medical University, Wroclaw, Poland; ^14^Universiy of Tampere, Tampere, Finland; ^15^First Department of Pediatrics, Center for Adolescent Medicine and UNESCO Chair on Adolescent Health Care, School of Medicine, National and Kapodistrian University of Athens, Athens, Greece; ^16^Department of Psychiatry, Koç University Hospital, Istanbul, Turkey; ^17^Private Psychiatric Practice, Sofia, Bulgaria; ^18^Belgrade University School of Medicine, Institute of Mental Health, Belgrade, Serbia; ^19^Hospital Clínico San Carlos, Madrid, Spain; ^20^Department of Psychiatry, Hospital Papa Giovanni XXIII-Bergamo (BG), Bergamo, Italy; ^21^Department for Affective Disorders, University Clinical Center of Serbia, Belgrade, Serbia; ^22^Department of Psychiatry, Referral Center for the Stress-Related Disorders, University Hospital Dubrava, Zagreb, Croatia; ^23^Mental Health Center “Prof. N. Shipkovenski”, Sofia, Bulgaria; ^24^Department of Pediatrics, University Hospital Center Mother Teresa, Tirana, Albania; ^25^Faculty of Medicine, Collaborative Antwerp Psychiatric Research Institute, University of Antwerp, Antwerp, Belgium

**Keywords:** maternity, parenthood, gender, training, workforce, migration

## Abstract

**Introduction:** Combining a successful career with family planning has become increasingly important in recent years. However, maintaining a relationship, deciding upon the optimal time for pregnancy and other family planning decisions can still be quite challenging, especially for junior doctors whose training is long and demanding. Currently, women form an important part of the medical workforce, and there is noticeable feminization in migration. However, little is known about the personal characteristics of junior doctors in Europe and how these play a role in their decision to migrate.

**Methods:** Survey of psychiatric trainees in 33 European countries, exploring how personal characteristics, such as gender, relationship status and parenthood, impact their attitudes toward migration.

**Results:** 2,281 psychiatric trainees in Europe took part in the study. In this sample, the majority of psychiatric trainees were in a relationship, but only one quarter had children, although there were variations across Europe. Both men and women indicated personal reasons as their top reason to stay. However, women ranked personal reasons as the top reason to leave, and men financial reasons. Single woman were the most likely of all subgroups to choose academic reasons as their top reason to leave. Interestingly, when women were in a relationship or had children, their attitudes toward migration changed.

**Conclusions:** In this study, a low number of psychiatric trainees in Europe had children, with differences across Europe. These findings raise awareness as to the role of parental conditions, which may be favoring or discouraging parenthood in junior doctors in different countries.

## Introduction

To combine a successful career with family planning has become increasingly important in recent years (1, 2). At the same time, maintaining a relationship, deciding an appropriate time for pregnancy and other family planning decisions are still quite challenging, especially for junior doctors whose training is long and demanding (3), following a time-intensive and competitive education path (4).

Although the medical landscape of Europe is complex, one study has shown that about half of the junior doctors had their first child in their early 30s once they engaged in registrar or specialist training posts, while 25% of women and 17% of men waited until becoming a consultant before becoming a parent (4). Doctors not only delay having children, but also tend to have fewer children than less educated people (5, 6). Furthermore, it has been described that the average family spends around a quarter of its income on childcare, or even more for more junior positions, such as medical students and foundation doctors (7). For doctors, family-work balance is influenced by many factors. Once identified, these factors can be optimized in order to promote better strategies for ensuring satisfaction both with family and work life (8). This concern is therefore critically important both to doctors as well as their employers (4, 9).

Currently, women form an important part of the medical workforce (10). In fact, over the recent years, there has been increasing discussion of “Feminisation” in the medical field, with the majority of medical students (11) and over half of the general practitioners (GP) being women (12). Importantly, more female than male doctors appear to choose what has been termed “people-orientated” specialties, which covers several non-surgical specialties, such as psychiatry (13–15). According to a survey of the National Health Service (NHS), female consultants chose psychiatry as it would enable them to pursue career paths compatible with family responsibilities (16). The “feminisation of psychiatry” (15) has been illustrated with women in more than half of the registrar posts (17).

There is also a “feminisation of migration” (18), which is not a new phenomenon (19). Women represent 48% of the 258 million international migrants worldwide (20). They outnumber men in developed countries, reaching 52%, while in developing countries they account for 46% of all immigrants (21). In fact, female migrants outnumber male migrants in Europe, North America, Oceania, Latin America, and the Caribbean (20). Besides, it has been observed that women have been progressively moving as independent or single migrants rather than as wives, mothers or daughters of male migrants (22). These cross-border movements are largely determined by economic factors (23), with women being part of the workflows, moving on their own to become the principal wage earners for their families (24). In addition to the economic factors explaining female migration, gender discrimination has proven to be of particular importance. Restrictions on the role assigned to women may act as a push factor, encouraging them to leave their home country (25). Women who do not feel treated with respect and dignity in their country express a stronger intention to emigrate, taking their skills elsewhere (19). On the other hand, it may also be exactly these restrictions what prevent them from leaving (26), such as economic or personal circumstances (27), along which gender imbalances might play a role (28). An outflow of human capital is generally troubling, with female losses being particularly costly, and having an impact in the wider family, including partners and children.

The research so far exploring migration and gender has reached contradictory findings. Some studies suggest that women who are young, single, highly skilled, employed, living in urban areas and in wealthier households have a higher chance of expressing an intention to move abroad. Equally, they have a higher chance of acting upon their desire to migrate (19). On the other hand, other studies argue that women's likelihood of acting upon their desire to migrate is lower than men's, referring to gender specific constraints, focusing on costs (28), health, basic finances and family obligations (27), as well as institutional hurdles related to migration and regulations restricting free movement of people (29).

Although there is a high rate of migration in search of work and personal fulfillment, the core family obligations and how family members negotiate their commitments seem to play a role in the decision to move (30). In fact, many women travel for the primary reason of keeping their family together (31), joining their partners in foreign countries and supporting their careers (32). Interestingly, some studies claim that having more family members and a larger number of children in the household, and therefore family obligations, appears to act as an additional incentive for women to move abroad (19).

While previous studies have focused on individual countries or occupations, little is known about the personal characteristics of junior doctors and how these play a role in their decision to migrate.

This paper aims to assess the personal characteristics of psychiatric trainees across Europe, such as relationship status, parenthood and living arrangements; and explore the impact of their personal characteristics in their experience and attitudes toward migration, including their reasons to stay or leave the country.

## Methods

### Study Design and Data Collection

This has been an international cross-sectional survey of psychiatric trainees in 33 European countries (EFPT Brain Drain Study), which received a favorable opinion by a National Ethics Committee in Switzerland. The questionnaire was circulated in each country in 2013–2014 by National Coordinators, either as an online survey (surveymonkey.com) and/or as paper questionnaires. The inclusion criteria were being a psychiatric trainee (defined as a fully qualified medical doctor enrolled in a nationally recognized specialist training program in psychiatry). More information about the survey is available elsewhere (33).

### Statistical Analysis

We analyzed the data using the Software Package for Social Sciences for Windows v. 22.0 (SPSS Inc. Chicago, IL). Descriptive statistics were used to report the frequencies and percentages for the categorical variables and the mean value with the standard deviation (SD) for the continuous variables. The set of questions on “migratory tendency” in the survey had a hierarchical structure based on participants' answers, whereby an affirmative answer at each question served as a gateway to the subsequent question. Hence, three hierarchical variables of steps of “migratory tendency” were created: “ever” considered leaving (yes/no); considering leaving “now”, recoded as a dichotomic variable (“strongly agree” or “agree” = yes, else = no) and taking “practical steps” (yes/no), describing an increasing disposition toward future migration.

Mixed effect logistic regression was used to assess the effect of the variables “gender”, “relationship status” and “parenthood” on migratory tendency, clustered by country of training, and adjusted for age. Differences in categorical variables such as the top reason to stay and leave the country between subgroups (variables “gender,” “relationship status” and “parenthood” and their interactions) were evaluated in contingency analysis with Pearson chi-square tests. Pearson correlation analyses and T-tests were used to evaluate the effect of independent factors on continuous dependent variables. Missing data were omitted on an analysis-by-analysis basis and valid percentages were reported.

## Results

### Sample Characteristics

#### Gender and Age

Data from 2,281 psychiatric trainees was obtained from 33 countries. The sample collected had more female respondents in the vast majority of the countries, except for Belarus and Finland (with 60.4 and 52% males respectively) as well as Ukraine, where the same number of female and male trainees responded (Table 1).

**Table 1:** Sample characteristics.

**Country**	**Response rate (%)**	**Mean age (years ± SD)**	**Gender (% female)**
Albania	100.0	31.9 ± 3.4	100
Belarus	37.3	26.2 ± 1.5	39.6
Belgium	79.4	27.1 ± 3.0	79.6
Bosnia and Herzegovina	82.2	33.5 ± 3.6	51.4
Bulgaria	72.4	31.9 ± 4.0	70.0
Croatia	60.0	32.9 ± 5.0	68.4
Cyprus	100.0	29.0 ± 1.6	100
Denmark	16.5	34.4 ± 6.4	82.8
Estonia	53.5	31.4 ± 7.4	90.9
Finland	21.2	36.5 ± 7.6	48.0
France	16.1	27.1 ± 2.3	64.8
Germany	16.0	33.6 ± 6.3	59.1
Greece	12.9	35.4 ± 4.1	56.8
Hungary	37.5	30.3 ± 5.6	68.4
Ireland	36.2	33.8 ± 5.7	64.6
Israel	15.0	33.4 ± 3.8	51.9
Italy	60.5	30.1 ± 3.0	64.1
Latvia	68.4	29.6 ± 5.7	58.3
Lithuania	73.8	28.5 ± 4.0	78.9
Malta	75.0	28.6 ± 2.6	80.0
Poland	41.3	29.2 ± 2.2	55.2
Portugal	53.9	28.7 ± 2.4	63.6
Romania	70.8	29.3 ± 3.9	72.5
Serbia	70.3	34.4 ± 2.8	61.5
Slovakia	31.3	31.5 ± 4.5	71.4
Slovenia	55.2	31.4 ± 5.7	63.9
Spain	38.0	30.6 ± 5.1	58.4
Sweden	50.0	36.1 ± 6.1	55.0
Switzerland	19.0	36.0 ± 7.9	70.7
The Netherlands	26.9	31.7 ± 5.6	71.2
Turkey	14.3	28.2 ± 2.7	72.8
United Kingdom	5.1	32.1 ± 4.8	63.2
Ukraine	44.4	30.5 ± 0.7	50.0

The mean age of the trainees in a relationship (31.2 ± 5.3y) did not differ from those single (30.8 ± 6.0y). However, trainees that were parents were on average 5 years older than trainees without children (35.2 ± 6.3 y vs. 29.8 ± 4.3 y; t22.0, *p* < 0.001).

#### Relationships, Parenthood, and Living Arrangements

**Figure 1:**
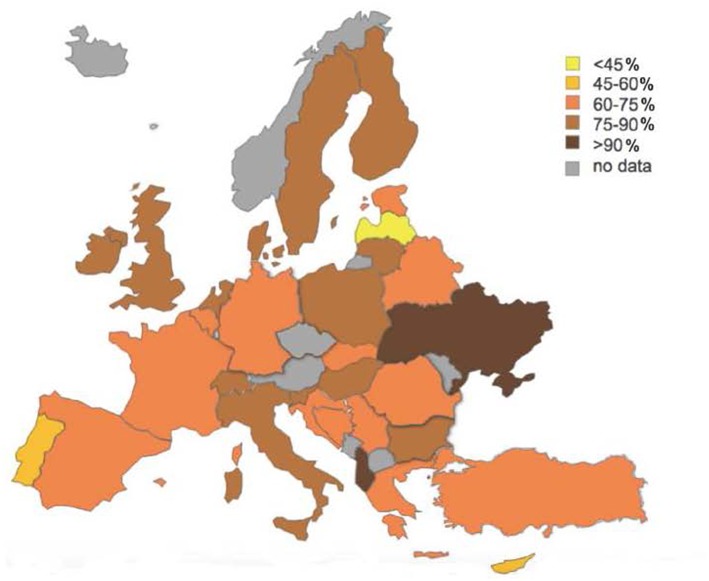
Psychiatric trainees relationship.

**Figure 2:**
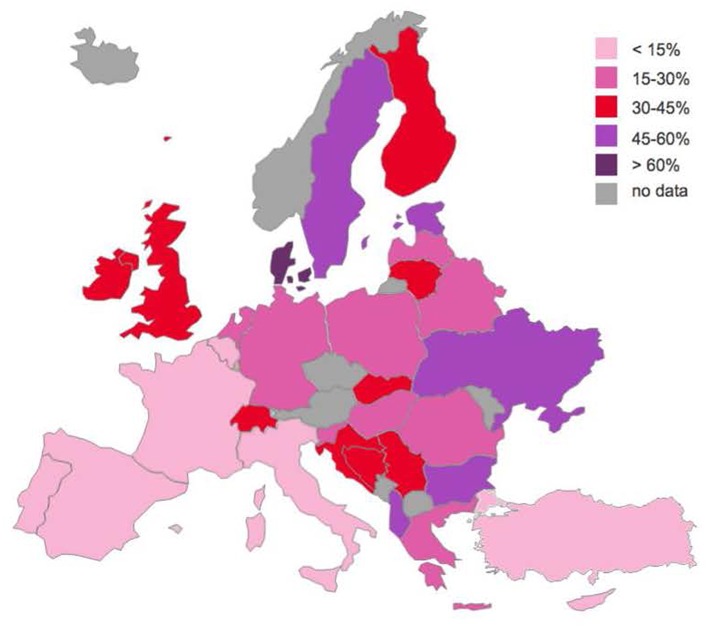
Psychiatric trainees who are parents.

The majority of the trainees were in a relationship (70.7% of men and 74.4% of women) and did not have children (75.6% of men and 73.4% of women), as described in Table 2. The differences across countries are illustrated in Figures 1, 2. In some Eastern countries, Albania (8.3%) and Ukraine (0%), only a few or none of the trainees were not in a relationship. On the other hand, Portugal (43.4%), Cyprus (50%), and Latvia (58.3%), had higher percentages of trainees that were single (not in a relationship).

**Table 2:** Family arrangements (relationship and children).

	**Men, *N* (%)**	**Women, *N* (%)**	**Total, *N* (%)**
**RELATIONSHIP STATUS**			
In a relationship	498 (70.7%)	1018 (74.4%)	1516 (73.2%)
Not in a relationship	206 (29.3%)	350 (25.6%)	556 (26.8%)
**CHILDREN**			
Yes	172 (24.4%)	364 (26.6%)	536 (25.9%)
No	532 (75.6%)	1004 (73.4%)	1536 (74.1%)
**NUMBER OF CHILDREN**			
1	95 (55.2%)	196 (54.0%)	291 (54.4%)
2	56 (32.6%)	131 (36.1%)	187 (35.0%)
3	20 (11.6%)	27 (7.4%)	47 (8.8%)
4	0 (0%)	8 (2.2%)	8 (1.5%)
5	1 (0.6%)	1 (0.3%)	2 (0.4%)
**DO CHILDREN LIVE WITH YOU**			
Yes	161 (93.6%)	353 (97.2%)	514 (96.1%)
No	11 (6.4%)	10 (2.8%)	21 (3.9%)

Regarding parenthood, 24.4% of male trainees and 26.6% of female trainees had children, most often one child (54.4%). The proportion of those with children varied across Europe from < 10% of trainees in Cyprus (0%), Portugal (6.1%) and Turkey (7.9%), to over 50% in Sweden (51.3%), Israel (57.7%), Albania (58.3%), Estonia (59.1%) and Denmark (62.1%) (Figure 2). These figures correlated significantly with the mean age (Pearson's r.585, *p* < 0.001), but not the gender distribution of trainees in a country.

The majority of the trainees who were parents lived together with their children (Table 2). However, for fathers (i.e., male trainees with children), children did not live with them twice as often as for mothers (6.4 vs. 2.9%), which was statistically significant [χ^2^ = 4.101, *p* = 0.043].

Regarding the living arrangements, the majority of trainees lived with their family or in rented accommodation (Table 3). However, there were variations across Europe, with countries like Albania and Malta, where all the trainees lived with someone else. In other countries, such as Portugal (40.4%), France (44.6%), Ukraine (50%) and Cyprus (75%), more trainees lived alone.

**Table 3:** Living arrangements (house sharing and ownership).

	**Men, *N* (%)**	**Women, *N* (%)**	**Total, *N* (%)**
**Living arrangements**			
Living alone	206 (29.4%)	334 (24.6%)	540 (26.2%)
Living with family	333 (47.5%)	694 (51.1%)	1027 (49.9%)
Living with friends	60 (8.6%)	103 (7.6%)	163 (7.9%)
Living with colleagues	18 (2.6%)	23 (1.7%)	41 (2.0%)
Living with roommates	1 (0.1%)	3 (0.2%)	4 (.2%)
Living with other people	83 (11.8%)	202 (14.9%)	285 (13.8%)
**Accommodation Arrangements**			
Owner of the flat/house	0 (0%)	3 (0.4%)	3 (0.2%)
Parent's house	66 (14.3%)	136 (16%)	202 (15.4%)
Rented	349 (75.9%)	643 (75.8%)	992 (75.8%)
Free accommodation	11 (2.4%)	31 (3.7%)	42 (3.2%)
Hospital accommodation	13 (2.8%)	17 (2.0%)	30 (2.3%)
Campus	11 (2.4%)	10 (1.2%)	21 (1.6%)
Other arrangements	10 (2.2%)	8 (0.9%)	18 (1.4%)

#### Effect of Gender and Parenthood on Migratory Tendency

In mixed effect logistic regression, trainees with children were less likely to have “ever” considered leaving their country (OR = 0.57, *p* < 0.001). Compared to men with no children, mothers (OR = 0.48, *p* < 0.001) were less likely to have “ever” considered leaving, with a similar trend observed for fathers, but this was not statistically significant (OR = 0.76, *p* = 0.185).

Out of those that have “ever” considered leaving, trainees with children were significantly more likely to be considering leaving “now” (OR = 1.49, *p* < 0.001). Moreover, compared to men with no children, mothers (OR = 1.63) were significantly more likely to consider leaving “now” (*p* < 0.001), with a similar trend observed for fathers (OR = 1.51), although this was not statistically significant (*p* = 0.058).

Gender was also significantly linked to taking “practical steps” toward migration, with women being less likely to take these practical measures (OR = 0.755, *p* = 0.042).

For those who had migrated to another country for more than 1 year, most of the trainees migrated alone (60.7%) (Table 4). More men than women migrated alone (68.4% vs. 59.1%), although these results were not statistically significant [χ^2^ = 2.727, *p* = 0.099].

**Table 4:** Migrating alone or with someone.

**Migrated**	**Men, *N* (%)**	**Women, *N* (%)**	**Total, *N* (%)**
Alone	80 (68.4%)	123 (59.1%)	210 (60.7%)
With someone	37 (31.6%)	85 (40.9%)	136 (39.3%)
With partner	17 (45.9%)	28 (32.9%)	49 (36.0%)
With parents	7 (18.9%)	17 (20%)	26 (19.1%)
With family	12 (32.4%)	26 (30.6%)	45 (33.1%)
With friends	0 (0%)	8 (9.4%)	8 (5.9%)
With others	1 (2.7%)	6 (7.1%)	8 (5.9%)

#### Reasons to Stay and Leave

When comparing the top reason given by trainees to stay or leave the country, between gender, relationship and parenthood status, differences appeared, as summarized in Table 5.

**Table 5:** Trainees' top reason to stay and leave their country (by gender, parenthood, relationship).

	**Men (%)**	**Women (%)**	**Single (%)**	**In a relationship (%)**	**No children (%)**	**With children (%)**
**TOP REASON TO STAY**						
Academic	19.8	13.9	24.5	12.7	18.6	8.5
Cultural	3.7	2.4	2.9	2.8	2.8	2.8
Financial	4.6	3.9	5.5	3.6	4.8	2.2
Personal	58.9	71.6	55.4	71.6	62.9	79.1
Political	1.4	0.7	1.5	0.7	1.0	0.8
Religious	1.6	0.8	1.2	1.0	1.1	1.1
Social	1.8	1.8	3.5	1.2	1.8	1.9
Work	6.4	3.1	4.7	4.0	4.7	2.8
Other	1.8	1.9	0.9	2.3	2.3	0.8
**TOP REASON TO LEAVE**						
Academic	18.7	17.2	23.9	15.4	20.5	10.1
Cultural	8.1	5.8	7.3	6.3	6.6	6.6
Financial	33.9	24.9	26.6	28.5	27.3	29.9
Personal	16.7	32.8	20.5	30.0	25.2	33.3
Political	3.7	3.4	3.0	3.7	2.7	5.7
Religious	1.2	1.6	2.7	1.0	1.5	1.6
Social	5.2	2.8	4.8	3.1	3.7	3.1
Work	11.1	9.9	10.9	10.1	11.0	8.5
Other	1.5	1.5	0.3	2.0	1.6	1.3

The main reason to *stay* differed significantly between men vs. women [χ^2^ = 26.9, *p* < 0.001], those in a relationship vs. single [χ^2^ = 45.9, *p* < 0.001], and parents vs. without children [χ^2^ = 37.2, *p* < 0.001]. Personal reasons prevailed in all subgroups, but those who were single and those without children (significantly) more often chose academic over personal reasons as their top reason to stay compared to those in a relationship or with children. Male trainees more frequently selected work as their top reason to stay compared to female trainees.

The top reason to *leave* differed significantly between men vs. women [χ^2^ = 40.9, *p* < 0.001], those in a relationship vs. those who were single [χ^2^ = 29.5, *p* < 0.001], and parents vs. those without children [χ^2^ = 28.1, *p* < 0.001]. Female trainees more frequently prioritized personal reasons, while male trainees' top reason to leave was financial. Trainees with children (significantly) more often indicated they would leave primarily for political reasons, and less often prioritized academic reasons compared to those without children.

When exploring the interaction between gender and parenthood or relationship status, the top reason to stay differed significantly between men and women with and without children [χ^2^ = 68.3, *p* < 0.001], and with and without relationship [χ^2^ = 85.0, *p* < 0.001].

**Figure 3:**
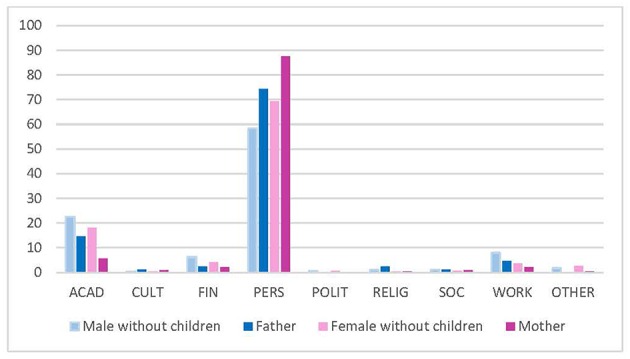
Top reason to stay (gender and parenthood). ACAD, academic; CULT, cultural environment; FIN, financial; PERS, personal; POLIT, political; RELIG, religious; SOC, social. Data reported in %.

Regarding parenthood, both male and female trainees prioritized personal over academic reasons to stay if they were parents (men without children 56.0%, fathers 67.3%; women without children 66.5%, mothers 84.3%) (Figure 3).

**Figure 4:**
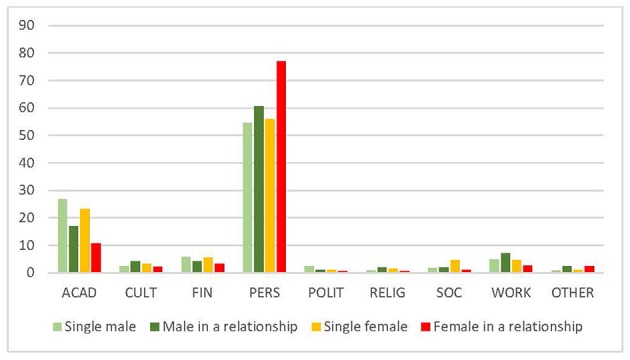
Top reason to stay (gender and relationship) (%). ACAD, academic; CULT, cultural environment; FIN, financial; PERS, personal; POLIT, political; RELIG, religious; SOC, social. Data reported in %.

Regarding the relationship status of trainees, both men and women who were in a relationship less frequently reported academic reasons as the top reason to leave, compared to those who were single. However, female trainees with a partner prioritized personal reasons to stay more often (76.9%) than single females (55.9%) and males, regardless of their relationship status (men in a relationship 60.1%, single men 54.5%) (Figure 4).

**Figure 5:**
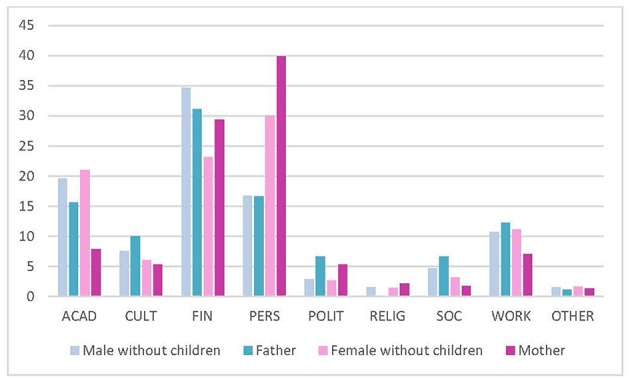
Top reason to leave (gender and parenthood). ACAD, academic; CULT, cultural environment; FIN, financial; PERS, personal; POLIT, political; RELIG, religious; SOC, social. Data reported in %.

The top reason to leave differed significantly between men and women with and without children [χ^2^ = 79.1, *p* < 0.001] and in or not in a relationship [χ^2^ = 83.8, *p* < 0.001]. While for men the top reason to leave (i.e., financial) did not significantly change depending on their parenthood status, women more often indicated they would leave primarily for personal reasons if they had children (women without children 30.0 vs. mothers 40.0%). In fact, mothers much more rarely chose academic reasons as their top reason to leave (7.9%) compared to women without children (21.0%) (Figure 5).

**Figure 6:**
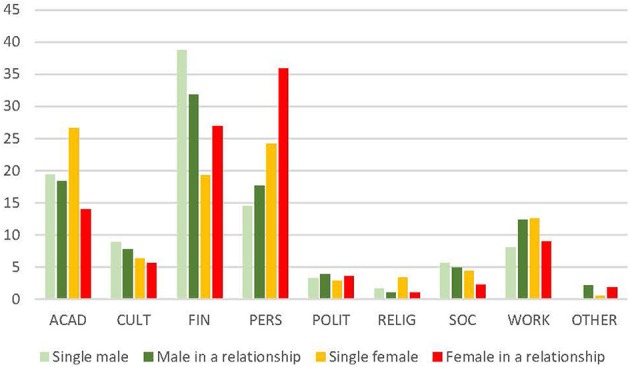
Top reason to leave (gender and relationship) (%). ACAD, academic; CULT, cultural environment; FIN, financial; PERS, personal; POLIT, political; RELIG, religious; SOC, social. Data reported in %.

Regarding relationship status, single female trainees more often indicated academic reasons as their top reason to leave (26.6%), compared to single male trainees (19.4%). Single men more often indicated financial reasons (38.7%), compared to single female trainees (19.3%). For female trainees in a relationship, the top reason to leave was personal (35.9%) (Figure 6).

## Discussion

### Key Findings

#### Profile of Psychiatric Trainees

These findings indicate that the majority of psychiatric trainees in Europe are in a relationship, and in some countries (such as Albania and Ukraine) only a small number of trainees are single. There are exceptions, however (as in Cyprus, Portugal and Latvia), where higher percentages of trainees are not in a relationship. Remarkably, only one quarter of the psychiatric trainees in Europe have children. Denmark is the country where the highest percentage of psychiatric trainees were parents, while Cyprus and Portugal were the countries where the majority of trainees did not have children. Trainees who are parents were older, and for the majority, their children lived with them. Of note, those who were fathers were twice as likely not to have their child living with them, compared to mothers.

#### Migratory Tendency

The majority of the trainees migrated alone, especially men. Furthermore, trainees with children were less likely to have “ever” considered leaving to another country, especially if they were mothers. Yet, mothers who “ever” considered leaving to another country, were more likely to consider it “now.” Still, women (regardless of whether or not they had children) were less likely to have taken practical steps toward migration.

#### Reasons to Stay and Leave

While both men and women indicated personal reasons as their top reason to stay in the country, they differed in their top reason to leave the country. Women mostly ranked personal reasons as driving factors in their decision to leave the country; whereas men mostly ranked financial reasons to leave. Overall, academic reasons seemed much more important factors in the decision to stay or to leave for single trainees without children. Interestingly, academic reasons were most frequently indicated as the top reason to leave by single women, and least frequently by women with children. This suggests that perhaps single women nowadays are quite career driven, searching for “status” or better opportunities abroad prior to establishing a family.

In particular, a point that deserves attention is the gender roles, as women seem to change their behavior more once they enter a relationship or have children, compared to men. Indeed, upon entering in a relationship or becoming mothers, women more often indicated they would stay and leave primarily for academic reasons, and less often for personal reasons, while men seemed much less affected by such a “relationship or parenthood effect.”

### Comparison With the Literature

The literature recognizes that medical doctors have fewer children than the general population (5, 34, 35), and these results show that psychiatric trainees are no exception. Progressing with training and continuing to develop professionally may be delayed by, or the desire to do so may decrease during maternity leave and time spent caring for young children. Moreover, frustrations, stress, lack of value and sense of fulfillment can also arise from reduced flexibility, the linear nature of most training programs and the pressure of working in services that are under-resourced. Equally, the long and variable hours of work required in medical practice, particularly in acute hospital service, where much of the training is undertaken, make it difficult to secure good quality and affordable childcare (4).

Still, these findings show variations in parenthood across Europe, which may be explained by different maternity and paternity leave conditions across countries, not only for doctors, but for the wider population of different European countries (36). For example, Denmark offers convenient parental conditions: a total of 52 weeks of paid parental leave, of which new mothers get 18 weeks of maternity leave (4 weeks fully paid before birth and 14 weeks after). During these 14 weeks period, fathers can also take two consecutive weeks off, and from that point, parents can split the additional 32 weeks, as they see fit. Other differences may arise for example with each trainee's year of training. For instance, in Romania it seems that several female trainees in their last year of training decide to give birth, getting up to 2 years maternity leave paid by the state before finishing their training. This (extra) time can assist them to become specialists and find a permanent position. In other countries, additional challenges to parents are posed by the local governments and infrastructures, with a lack of childcare facilities that would allow working parents to accommodate their newborns and children. In addition, childcare is considerably costly or cannot allow for convenient dropping off or collection times around working hours (37). Thus, parents are forced to consider reducing their working hours, to defer their training for a period or to continue working with implemented pauses.

In a previous paper published from this study, which analyzed the overall reasons for all psychiatric trainees to stay or leave the country (regardless of gender, relationship status, and parenthood), for all trainees the top reason to stay in the country was personal, followed by academic reasons, whereas financial and personal reasons were the top reason for trainees to leave the country (33). This suggested that income had a major impact on the migratory tendency of trainees, while acknowledging the existence of other factors that play a role in the decision of some doctors to migrate (termed “atypical migration”). However, it did not further explore the role of demographic characteristics on trainees' decision-making to migrate.

In fact, in most of the previous literature, female migration has been linked to their role and status within the family, defined in relationship to their male partners (18). Yet, the demographics of those who elect to migrate has changed favoring women and younger individuals. This may be explained by the greater female presence in education (38) and an increase in international migration of youth seeking better education (39). In reality, multiple push and pull factors seem to influence women's migration, which include complex interactions between economic, social and family factors, as well as employment and healthcare. Since in many societies such rights are withheld from women, migration to economically and educationally more open societies often can help improve their personal situations and employment opportunities. In fact, one feature of the economic impact of globalization is the increase in the transnational migration of women seeking work (40).

Equally, parenthood raises specific concerns with gendered subjectivities, experiences of care and parental obligations (41). Previous research has explored family migration decisions involving negotiations on care over time, the reasons to search for a life abroad, gendered patterns, and the moral dimensions of migration decision-making as a measure of “good” parenting (41).

It seems that on average, one-third of women considering to migrate have family or friends abroad, and the number increases significantly for those actually taking “practical steps” toward migration (19). There is therefore a strong impact of social networks on both migration intentions and further migration behavior (42). In fact, research on family and child migration (43), exploring the role of social networks to understand migration patterns and processes (44), has showed that some of these movements occur to keep groups intact (45). Although previous research has raised awareness on the positioning of children within family migration (46) and the lack of acknowledgment of the direct and indirect role that children may have on migration decisions (47), that has not been the focus of this paper.

In any case, the decision to migrate seems to involve several steps, not all of them observable and measurable (48). Earlier studies have already recognized the distinction between migration intentions and actions (49, 50). In particular, a study comparing the intention to migrate and the subsequent migration behavior of woman, found that women's probability to carry out their migration plans is systematically lower than men's and concluded that women's unrealized migration plans are due to gender-specific costs and constraints (28). Our findings concur with this, as we found women less likely to take practical steps toward migration. Knowing what drives the intention to migrate allows one to assess the subpopulation who would consider moving abroad, which in itself yields interesting insights into future migration trends (29, 49).

### Strengths and Limitations

To the best of our knowledge, this has been the first paper looking at the influence of gender and family arrangements on the migratory tendency of junior doctors. It has also been the study with the largest sample size of psychiatric trainees in Europe (n = 2281) and including more countries (*n* = 33) (33). Despite its originality, it has several limitations.

First, sampling rates varied within countries, with some countries having lower response rates, introducing selection bias in the response rate. Additionally, as a self-report questionnaire, it is subject to recall and reporting bias, as well as social desirability bias. Importantly, the findings refer to different types of migratory tendencies (“ever”, “now,” and “practical steps”), and it is unclear which parameter optimally assesses the intention to migrate. Furthermore, in the top reasons to stay and leave, eight options were provided for trainees to choose from (academic, cultural, financial, personal, political, religious, social, and work related), with an explanation of what each would cover. For example, the option called “personal” reasons, would include reasons linked with their partner, children and family. Therefore, we cannot infer to which aspect in particular they were referring. Moreover, relationship status, parenthood and living arrangements are not long-term characteristics but rather a current and interchangeable status of a person. Therefore, the reported characteristics are contingent on the time when participants responded to this survey.

Finally, as the study has not been developed primarily to answer questions related to parenthood and work-life balance, it does not provide us with all the relevant country-specific information (such as differences in infrastructure for parents, parental leave, workload etc.), which could be used to explain how migration status is related to family planning. Nevertheless, the study provides insight into the family and relationship status of a large number of trainees, which is one of the strengths of this paper.

### Relevance of the Findings and Implications for Practice, Policies, and Research

This study provides a valuable description of the profile of psychiatric trainees across Europe and the impact of their personal characteristics and family obligations on their decision and reasons to migrate.

These findings can be used to support trainees who are (or want to be) parents. To improve the conditions of junior doctors who want to establish a family during their training period, careful consideration is required to the working conditions and socio-cultural challenges of being a working parent, acknowledging the gendered experiences and practices of parenthood, where typically a large burden falls on women. Migration can provide new opportunities for women when they migrate on their own, or jointly with their partners, leading to potential improvements in their lives.

In fact, the reasons expressed by mothers, taking into consideration mostly personal reasons for their migration wishes, encourage us to reflect upon the fact they do not want to be “absent mothers” leaving their children behind (51, 52). Equally, this raises awareness of parental care concerns when parents decide to migrate while their children, at least initially, stay in their home countries in the care of others.

In particular, these findings can be used to support parental leave, to avoid what is a reality for many: not being able to progress professionally/clinically or having their projects taken over, while on maternity leave. Usually trainees who become mothers have a career break, which in some countries means losing opportunities compared to their male counterparts, in a key period of their professional development. This situation might influence their decision toward migration.

To better understand this delicate reality, future research should also be qualitative, further exploring the reasons and motivations for junior doctors to migrate. This would enable us to further understand the ways in which relationship status, parenthood and childcare may be involved in migration decision-making process, and to learn further, for those who did move, their experience and life abroad.

Equally, future research should explore the integration of doctors who are parents in their host country. We know that migrants can experience stress and anxiety due to the loss and separation from their established home, social, and cultural environments. Their social integration in new host settings may be equally limited by their initial lack of educational, occupational, and social experiences. Perhaps skilled doctors integrate themselves differently or perhaps have better chances of adapting to a new environment.

Finally, these results were collected at a time of free movement in the European space. Yet they call for further research on family migration on other professionals within the EU, where the “free movement” is exposed to less scrutiny, further exploring mobile family practices and obligations. In addition, future studies should also explore these migratory intentions in non-free movement spaces.

## Conclusions

In our study, the majority of the psychiatric trainees in Europe were female, single, without children, living with family or renting a house.

These results show that gendered family configurations and parental responsibilities are crucial for deciding whether to stay or leave the country. Although women weighted more heavily personal reasons in their decisions to migrate, particularly single women ranked academic reasons as the most important driver for them to leave the country. Trainees who were parents were more likely to report personal reasons to stay and those without children were more likely to stay for academic and work reasons. As for leaving, trainees who were parents were more likely to leave for personal and financial reasons, and those who did not have children were more likely to leave for academic and work reasons.

## Contributors

The following colleagues have also acted as (or have collaborated with) the National Coordinators of this study, supporting the Chief Investigator and International Coordinator (Mariana Pinto da Costa) in the data collection in their countries: Uladzimir But-Husaim (Belarus), Andreas Hoff and Celina Skjodt (Denmark), Doris Madisson (Estonia), Adrien Pontarollo and Thomas Gargot (France), Edina Kiss (Hungary), Ben Amit (Israel), Nikita Bezborodov (Latvia), Ilona Uleviciute-Belena (Lithuania), Marija Farrugia (Malta), Katarina Ceranic (Slovenia), German Strada (Spain), Gina Necula, Ilinca Mihailescu, Cezar Oanea, Luiza Voicila, Raluca Tirintica, Anca Popescu, Marinela Hurmuz, Oana Cornutiu and Radu Oroian (Romania), Tove Mogren (Sweden), Jelly Kuiters (The Netherlands), Kevin Holmes (United Kingdom), and Orest Suvalo (Ukraine).

## Author Contributions

MPC designed the study, coordinated and supervised the study group, the statistical analysis and drafted the manuscript. LD contributed to the statistical analysis. All authors contributed to the collection of data, read and approved the final manuscript.

### Conflict of Interest Statement

The authors declare that the research was conducted in the absence of any commercial or financial relationships that could be construed as a potential conflict of interest.
